# Experimental Study on Silt Soil Improved by Microbial Solidification with the Use of Lignin

**DOI:** 10.3390/microorganisms11020281

**Published:** 2023-01-20

**Authors:** Yongshuai Sun, Xinyan Zhong, Jianguo Lv, Guihe Wang

**Affiliations:** 1 College of Water Resources & Civil Engineering, China Agricultural University, Beijing 100083, China; 2 School of Engineering and Technology, China University of Geosciences, Beijing 100083, China

**Keywords:** MICP, lignin, silt, unconfined compressive strength test, dynamic triaxial test

## Abstract

At present, in the field of geotechnical engineering and agricultural production, with increasingly serious pollution an environmentally friendly and efficient means is urgently needed to improve the soil mass. This paper mainly studied the microbial induced calcium carbonate precipitation (MICP) technology and the combined effect of MICP technology and lignin on the improvement of silt in the Beijing area. Through unconfined compressive strength and dynamic triaxial test methods, samples improved by microorganisms were studied to obtain the optimal values of cement concentration and lignin under these two test schemes. The results show that after the incubation time of *Sporosarcina pasteurii* reached 24 h, the OD600 value was 1.7–2.0 and the activity value (U) was 930–1000 mM ms/min. In the unconfined static pressure strength test, after MICP treatment the optimal concentration of cementitious solution for constant temperature and humidity samples and constant-temperature immersion samples was 1.25 mol/L. The compressive strength of the constant temperature and humidity sample was 1.73 MPa, and the compressive strength of the constant-temperature immersion sample was 3.62 Mpa. At the concentration of 1.25 mol/L of cement solution, MICP technology combined with lignin could improve the constant temperature and humidity silt sample. The optimal addition ratio of lignin was 4%, and its compressive strength was 1.9 MPa. The optimal lignin addition ratio of the sample soaked at a constant temperature was 3%, and the compressive strength was 4.84 MPa. In the dynamic triaxial multi-stage cyclic load test, the optimal concentration of cementation solution for the constant temperature and humidity sample after MICP treatment was 1.0 mol/L, and the failure was mainly inclined cracks. However, in the condition of joint improvement of MICP and lignin, the sample mainly had a drum-shaped deformation, the optimal lignin addition ratio was 4%, and the maximum axial load that the sample could bear was 306.08 N. When the axial dynamic load reached 300 N, the strain accumulation of the 4% group was only 2.3 mm. In this paper, lignin, an ecofriendly material, was introduced on the basis of MICP technology. According to the failure shape and relevant results of the sample, the addition of lignin was beneficial for the improvement of the compressive strength of the sample.

## 1. Introduction

Gravel, sand, silt, and mudstone occupy most of the strata in the Beijing area. Based on the “Beijing Multi-parameter Geological Stereoscopic Survey” [1], it can be seen that silt, sand, and gravel are the most representative soil layers in Beijing. A defect in the engineering properties of silt is that it is prone to liquefaction [2,3] and other problems. In actual engineering, it is necessary to improve such poor soil. In recent years, the use of microbial mineralization for soil improvement technology (MICP) has attracted the attention of the academic community. Compared with traditional improvement methods, the main advantages of MICP are a simple construction method [4,5], low costs [6,7], environmental friendliness [8,9], easy control of the reaction process [10], easy penetration into geotechnical materials [11,12], and good durability [13,14]. MICP methods mainly include urea hydrolysis [15,16], denitrification reactions [17,18], ferric reduction [19], and sulfate reduction [20,21]. Among these, the hydrolysis of urea can quickly produce carbonate ions so the generated calcite can be cemented between soil particles to produce a bonding effect and improve the overall strength. The factors affecting the formation of calcium carbonate can be divided into five parts: the properties of the cemented soil particles [22,23,24]; the type, quantity, and activity of microorganisms involved in the reaction [25,26,27]; the concentration, type, and pH of the cementitious solution [28,29,30,31]; the content and uniformity of the calcium carbonate [32,33,34]; and other environmental factors, including the mixing method of bacteria, the cementation reagents and soil, the nutrients required for microbial growth and metabolism, and Ca^2+^ [35,36,37,38,39].

Microbial mineralization is an ecofriendly technology that can be used to carry out large-scale and long-distance soil reinforcement. The experimental study of using microorganisms to improve unfavorable soil makes it possible to apply this method to engineering. Scholars have used MICP technology to improve unfavorable soil to varying degrees, including sand [40,41,42], silt [43,44,45], loess [46,47,48,49,50], expansive soil [51,52,53,54], and mucky soil [55,56], using this method, and the unconfined compressive strength and cohesion of the soil are significantly improved, which can be used in engineering practice. In the field of geotechnical engineering, the research results of MICP on soil improvement can be used for foundation reinforcement [57,58,59], earth dam reinforcement [60], wind prevention, sand fixation [61], reservoir bottom anti-seepage, and anti-seepage curtains [42]. In the field of agricultural production and construction, MICP technology also plays a beneficial role in improving soil mass, such as water and soil conservation, the prevention of water and soil loss [62], and stability improvement in agricultural water conservancy projects [63] and agricultural buildings [64], which is conducive to planting and production activities and also ensures safety. Testing the mechanical properties of the cemented samples can not only measure the quality of the cementation effect but can also determine the feasibility of using the MICP technology based on urea hydrolysis to improve the soil. The unconfined compressive stress–strain curve can be obtained through the unconfined compressive strength test; the triaxial compression stress–strain curve can be obtained from the triaxial compression test, which generally has three stages: linear elasticity, yield, and ductile flow. Scholars have added soil conditioners to improve soil strength in their research on soil improvement experiments, including poly-Lys [65], foaming agents [66,67], reactive magnesium oxide [68], bentonite [69,70], cement [71,72,73], fiber [74,75], polyacrylamide [76,77], and quicklime [78,79,80]. The improvement effects using different contents of modifiers have been discussed, and the overall strength has improved; however, the production of soil modifiers such as cement and lime causes great pollution to the environment. Therefore, this study introduces lignin, a natural, efficient, and environmentally friendly soil conditioner, to stabilize soil based on environmental friendliness.

The improved effects of different concentrations of modifiers on soil strength were studied. Previously used solid modifiers such as cement and lime are hazardous to the environment; thus, this study used lignin, an environmentally friendly soil conditioner, to stabilize the soil. The enhanced effects of *Sporosarcina pasteurii* mixed with lignin on silt soil solidification in the Beijing area were analyzed using a growth curve and the incubation time of MICP. This helped us obtain the optimal value of cementitious liquid or lignin when MICP technology or MICP mixed with lignin was used to improve silt.

## 2. Experimental Materials and Methods

### 2.1. Experimental Materials

#### 2.1.1. *Sporosarcina Pasteurii*

The cultivation of *Sporosarcina pasteurii* is the first step in the study of microbial mineralization (MICP). In this study, the change rule of *Sporosarcina pasteurii* concentration and urease activity was explored using different cultivation times, and based on this, the optimal cultivation time of *Sporosarcina pasteurii* during sample preparation was selected. The strain was *Sporosarcina pasteurii* with ATCC 11859. The experiment used a nutrient broth liquid medium, including (g/L) 10 g of peptone, 3 g of beef extract powder, and 5 g of sodium chloride. We inoculated the bacterial liquid composed of bacterial species and liquid medium into a constant-temperature culture shaker and incubated it at 30 °C and 200 rpm for 1–2 days.

**Figure 1:**
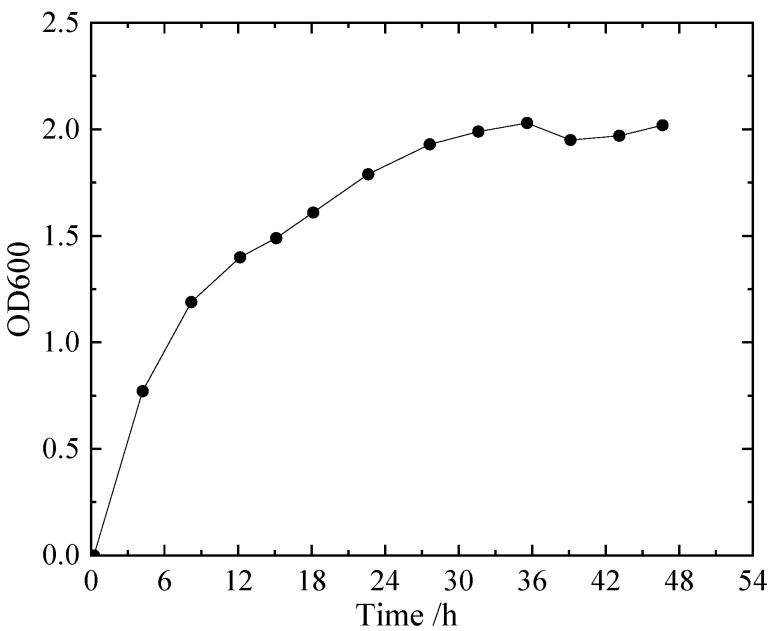
Growth curve of *Sporosarcina pasteurii*.

The OD600 value of the solution (the absorbance of bacteria at a wavelength of 600 nm) was measured using UV spectrophotometry to determine the concentration of bacteria. The growth curve of *Sporosarcina pasteurii* obtained from the experiment is shown in Figure 1.

During the proliferation of *Sporosarcina pasteurii*, there was an enzyme in the metabolite, which was urease; this can catalyze the hydrolysis of urea into ammonia and carbon dioxide. The conductivity change value of 1 ms/min corresponds to the urea hydrolysis amount of 11 mM. The relationship is as follows:

(1) U=SKA

In the formula, *S* is the dilution ratio of the tested bacterial solution; *K* is the measured conductivity change value (ms/min); *A* is a constant, taking *A* = 11.11; and *U* is the measured urease activity value (mM ms/min).

**Figure 2:**
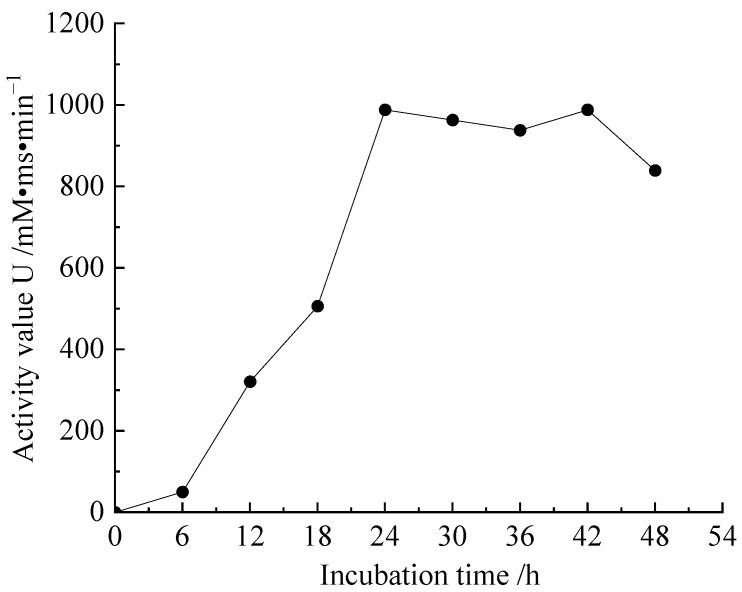
Variation diagram of bacterial activity.

The tested urease activity curve is shown in Figure 2. In this study, the dilution ratio of the bacterial solution was 10, that is, *S* (dilution time of tested bacterial solution) = 10 in the activity calculation formula, and the measured conductivity change value (k) was the average value of 5 min after adding the bacterial solution. As the number of bacteria increased, urease also increased, so the measured urease activity also increased, reaching a peak of 988 mM ms/min at the 24th h. Combined with the growth curve and activity curve of the cultured bacteria in this test, it can be seen that *Sporosarcina pasteurii* with a suitable concentration and high activity could be obtained within 24 h, so the culture time for the bacteria selected for the follow-up test was within 24–34 h.

#### 2.1.2. Silt

The soil used for the test was taken from a layer 2–3 m below a construction site in Beijing, which was sandy silt. The standard penetration N was in the range of 5~9, and the density was slightly dense. The soil used for the test was naturally dried in the room and passed through a 1.0 mm sieve before being used for sample preparation. The following basic physical properties were studied on the obtained test soil: the liquid-plastic limit and plastic index, the particle size gradation curve, the specific gravity, the air-dried moisture content of the test soil during sample preparation, and the density of the sample. The relevant results are shown in Table 1 and Table S1.

After calculation, the non-uniformity coefficient of the test soil was Cu = 8.25 > 5, and the curvature coefficient was 3 > Cc = 1.485 > 1, which indicated well-graded soil.

#### 2.1.3. Preparation and Maintenance of Samples

The preparation method used for the mechanical test sample was the sampling method: weigh a certain mass of soil; add the solution; mix evenly; put it into the sampler 3–5 times; operate the hammer 30 times each time; after the last hammer, shovel off the soil that it is higher than the mold; flatten the sample; and then demold. The soil, bacteria, lignin, and cementation reagent were mixed evenly. Then, the sample was prepared, so this sample preparation method is called the mixing method. The test included two sizes of sample makers: 39.1 mm × 80 mm and 50 mm × 100 mm. Small specimens were used for the unconfined test, and large specimens were used for the dynamic triaxial test. The material amounts and quantities of the samples are shown in Table 2 and Table S2.

There are two main ways to cure samples: constant temperature and humidity curing and constant-temperature immersion curing. The main production process for the sample using constant temperature and humidity curing was as follows: after the soil was dried and screened indoors, three samples were made in each group, and the solution was as follows: solution (cementation reagent and bacterial liquid) = 1000 g:200 mL ratio. Before sample preparation, the soil and bacterial liquid were mixed evenly, the cementation reagent was added to mix, and the sample could be prepared. The sample was cured in a curing box at 30 °C for 7 days and then dried at a low temperature. The mass of each sample after curing was 382 ± 5 g (the weight range of the sample used for the dynamic triaxial test); if the density change was within 1.31%, it was determined that the obtained sample conformed to the “Geotechnical Test Method Standard” in terms of density difference. The samples of the constant-temperature immersion method were consistent with the constant temperature and humidity sample preparation process; the main difference was the maintenance phase. The constant temperature and humidity method meant that the prepared samples were cured under suitable temperature conditions, while the constant-temperature immersion method involved putting the prepared samples into a cementation reagent with a pH between 7.3 and 7.4 for curing, and the curing time was 7d.

### 2.2. Test Equipment

#### 2.2.1. Unconfined Compressive Strength Test Equipment

**Figure 3:**
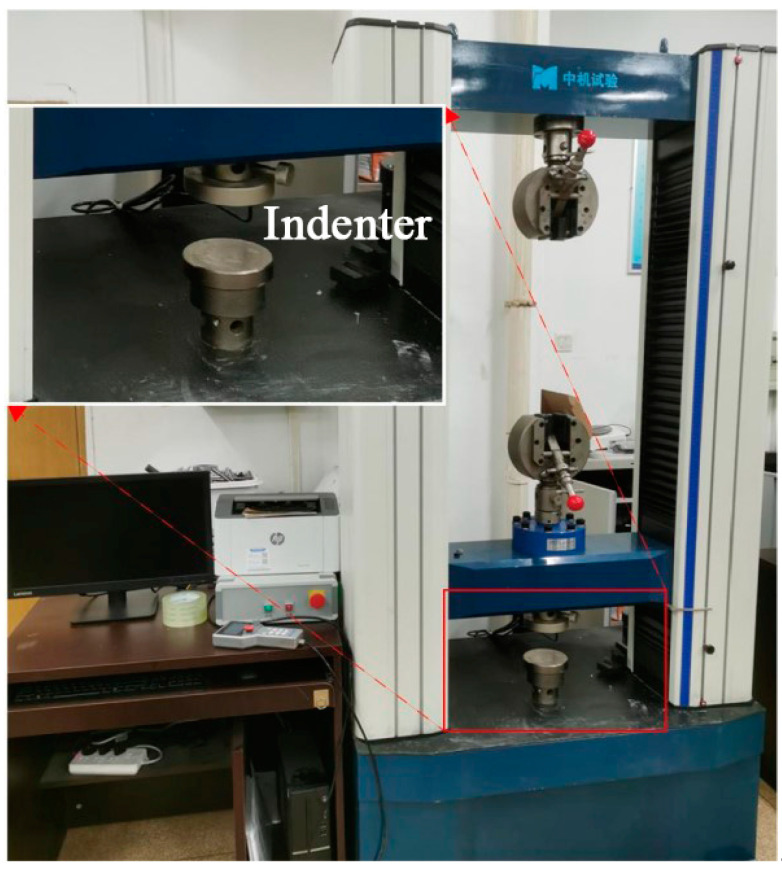
Universal testing machine.

Unconfined mechanical property experiments were carried out on the samples using the universal testing machine shown in Figure 3. The specimen used for the test was cylindrical. Under the action of no confinement stress, the maximum axial stress (q_u_) was measured, which was the compressive strength of the specimen. The universal testing machine used in this test was the electronic universal material testing machine RGM-100kN. The specimens were crushed under the action of axial pressure, and the displacement control method was adopted at 0.05 mm/min. There were two curing methods: the constant temperature and humidity curing method and the constant-temperature-soaking curing method, both of which require drying at a low temperature of 60 °C to obtain samples.

#### 2.2.2. Unconfined Compressive Strength Test Equipment

To study the mechanical characteristics of the improved silt under dynamic loads, a certain dynamic load was applied to the improved samples; in this way, the performance differences of the modified samples under dynamic loading were studied. After curing, a sample was directly subjected to a dynamic triaxial test under the condition that it was not consolidated and did not drain, and the confining pressure was 50 kPa; the sample did not need to be dried at a low temperature. The KTL dynamic triaxial system was used to conduct dynamic triaxial tests on the samples. The applicable sample size was 50 mm × 100 mm. Under the condition of a confining pressure of 50 kPa, axial loads including a sine-type wave, a frequency of 1 Hz, an amplitude of 60% of the central load, and 10 vibration times were applied in stages until the specimen failed or the strain reached 6%; the axial loads applied step by step were 50 N, 100 N, 200 N, 300 N, 400 N, and 500 N.

## 3. Test Scheme for Improving Silt with Microorganism Lignin

### 3.1. Optimal MICP Cementation Solution Concentration Test Scheme

In order to study the effect of *Sporosarcina pasteurii* on the improvement of silt soil, the bacterial liquid cultured between 24 and 32 h was selected for mineralization. In the experiment, the water used for culturing bacteria in soil was pure water, and anhydrous calcium chloride and urea were used to prepare a cementation solution. The following were also used: anhydrous calcium chloride with a mass fraction of 110.98 g/mol in the form of white powder, granules, or frit and urea with a mass fraction of 60.06 g/mol in the form of colorless crystals or white crystalline powder.

The culture of *Sporosarcina pasteurii* is described in Section 2.1. The soil used in the experiment was naturally air-dried indoors, and we sieved the resulting silt. In this study, the effects of five different concentrations of cementing solution on the microbial mineralization of *Sporosarcina pasteurii* were investigated, wherein the concentration of zero was the experimental control group. The substance contents of different cementation solution concentrations are shown in Table 3.

### 3.2. MICP and Lignin Joint Test Scheme

Lignin is a complex organic polymer. It is a biopolymer with a three-dimensional network structure formed by the interconnection of three phenylpropane units through ether bonds and carbon bonds. Lignin is consistent with the cell surface properties of *Sporosarcina pasteurii*. After being dissolved in water, it has negative ions, which allows it to easily attract cationic Ca^2+^, which in turn promotes its aggregation and crystallization on the surface.

The lignin (>99.5%, soluble in water) used in this experiment was purchased from Hefei BASF Biotechnology Co., Ltd. (Hefei, China). First, a preliminary study was carried out regarding the effect of lignin on improving silt, and the effect of lignin addition on the performance of silt was mainly studied using the different ratios of lignin addition. According to the research of most scholars on lignin-improved silt, the optimal addition ratio of lignin varies in different regions and different types of soils, e.g., lignin addition at percentages of 3%, 6%, and 12%. Therefore, the unconfined compressive test was used to determine the appropriate range of the addition ratio of the improved silt. For the blank control group, no lignin was added, and for the 3%, 6%, 9%, 12%, and 15% groups, those with addition ratios of 3%, 6%, and 12% will be fully covered below. Through the unconfined compressive strength test, the maximum axial load (Fmax) ranges that the samples with different lignin addition ratios could bear were obtained, as shown in Table S3.

After the preliminary study regarding the effect of lignin, it was found that, for the soil used in this experiment, the proportion of added lignin should be within 6%. This was verified by the results of the MICP scheme, as the cementing effect was best when the concentration of the cementation solution was 1.25 mol/L. Therefore, in this test plan, the cementation solution at a concentration of 1.25 mol/L was selected to study the effect of MICP technology combined with lignin at different concentrations, such as 1.5%, 3%, 4%, and 5% (M-L plan), to improve silt. The research was mainly conducted through unconfined and dynamic triaxial tests.

## 4. Results and Discussion

### 4.1. Analysis of Unconfined Hydrostatic Strength Test Results

#### 4.1.1. Analysis of the Improvement Effect of the M Scheme

For the samples cured at a constant temperature and humidity after mixing, we used this method to test the performance effect of improving silt via the mineralization of *Sporosarcina pasteurii*. We mainly considered and explored the optimal cement concentration value from the perspective of different cement concentrations and set groups with different cement concentrations (n represents any concentration in the set group), as shown in Figure S1a. In Figure S1, from left to right (a–f), these are the blank group and the 0.5 mol/L, 0.75 mol/L, 1.0 mol/L, 1.25 mol/L, and 1.5 mol/L groups. We observed the failure shape of the sample under the indenter, mainly through cracks that appeared above and below. The maximum load that could be endured after the failure of the sample dropped rapidly, which belonged to brittle failure. There were generally two cases for the development of sample cracks. One was that the tiny cracks generated in the middle penetrated both ends of the sample, and the other was the development of side cracks from the upper and lower end faces. These two kinds of cracks eventually caused the sample to break into two parts, and because the two end faces of the sample were restrained by the indenter, it generally remained intact, while the fracture surface and the two end surfaces formed a Z-shape or an I-shape.

**Figure 4:**
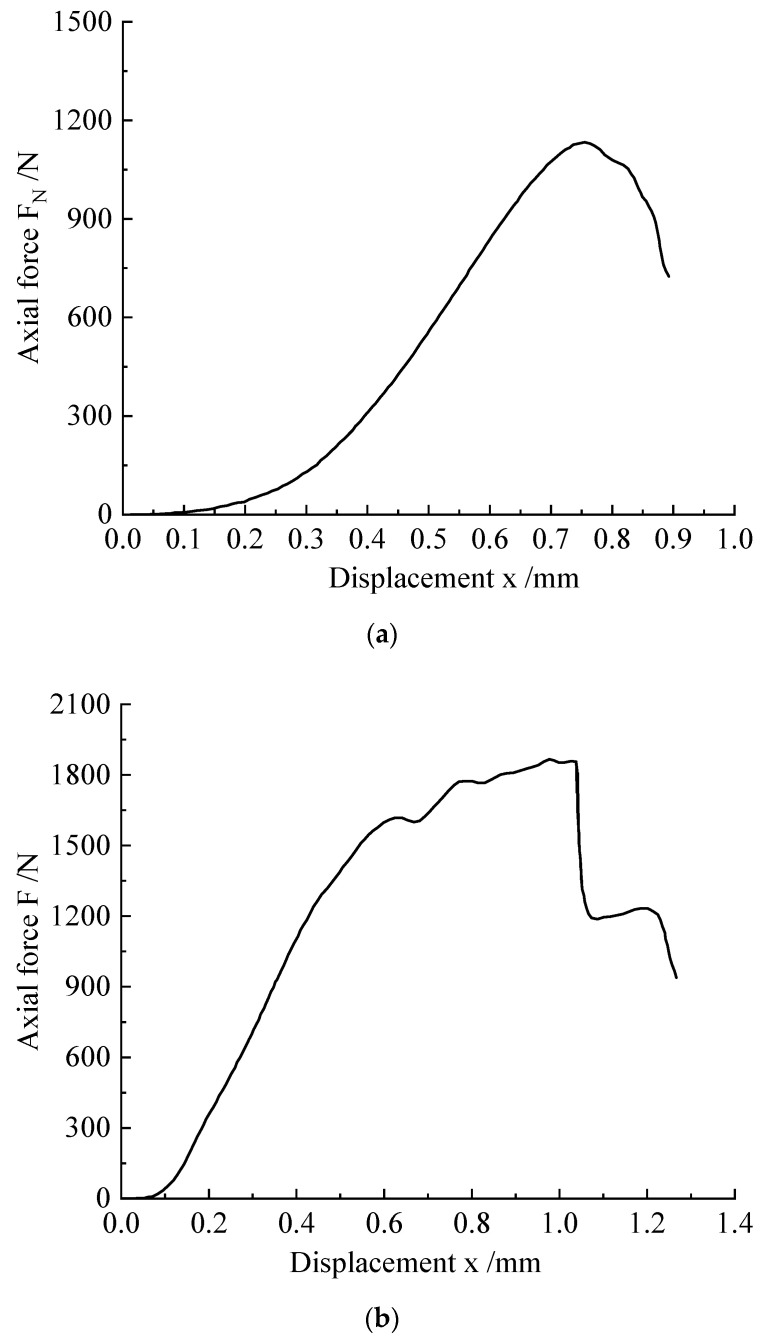
The axial force–displacement curves of M scheme combined with constant-temperature samples with different cement concentrations. (**a**) Axial force–displacement curve of the constant-temperature sample with an M-0.5 mol/L cementation solution. (**b**) Axial force–displacement curve of the constant-temperature sample with an M-1.25 mol/L cementation solution.

We took the force and displacement curves of the modified sample mixed with 0.5 mol/L of *Sporosarcina pasteurii* as an example (Figure 4a). The compression failure of the sample was generally divided into four stages. In the crack closing stage, under the action of low pressure, the displacement of the sample increased. In the elastic stage, the slope was roughly constant, and the force and displacement had a linear relationship. In the development stage of plastic deformation, when the axial force reached a certain value, the sample underwent plastic deformation, and the deformation became more obvious with the increase in the force. In the cracking failure stage, the sample was damaged. The ratio of the corresponding axial compression value (Fmax) during the compression process to the compression section of the specimen was the compressive strength of the specimen.

**Figure 5:**
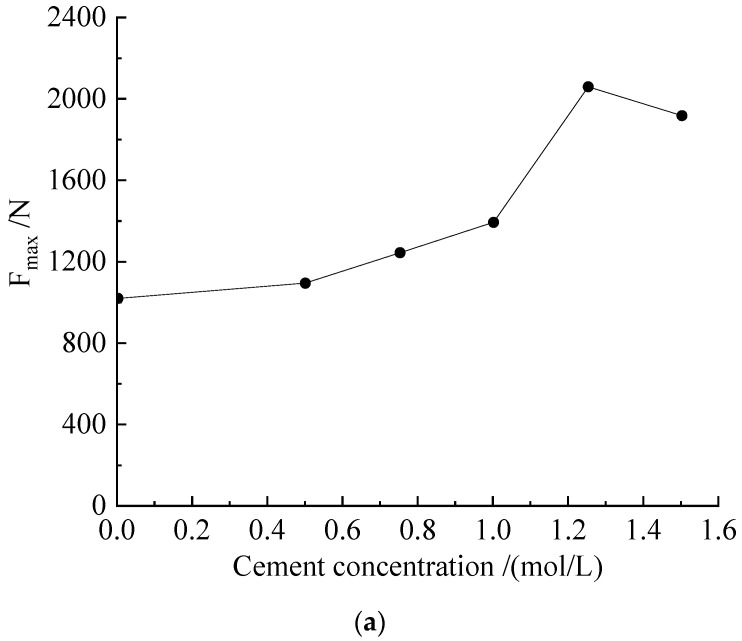

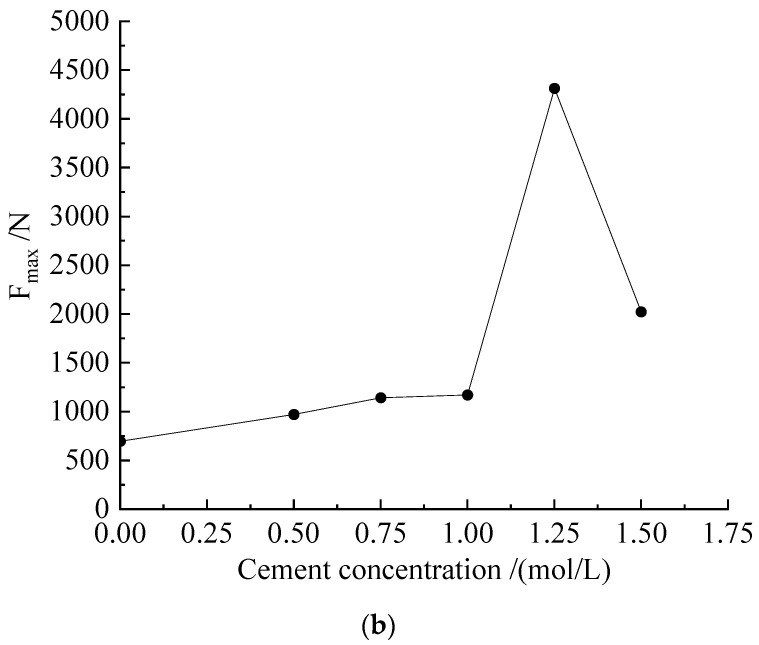
Axial force (F_max_)–concentration curves of different samples in scheme M. (**a**) Axial force (F_max_)–concentration curve of cementation solution of constant-temperature sample in M scheme. (**b**) Axial force (F_max_)–concentration curve of cementation solution for soaked samples in scheme M.

Another typical curve change also had four stages; the difference from the above curve is that, after reaching the maximum value of the axial force, there was a drop phenomenon of the axial force. As an example, the force and displacement curves of *Sporosarcina pasteurii* mixed with a 1.25 mol/L cementation solution are shown in Figure 4b. Compared with the mixing of the 0.5 mol/L cementation solution and compared with the previous curve, there was a decrease in the axial force value before the maximum value; such fluctuations are generally caused by local failure of the specimen or the development of microcracks. When the tiny cracks in the sample gradually developed until they penetrated the sample, the axial force drop phenomenon, shown in Figure 5a, appeared. During the test, the sound of the sample destruction could be heard. Figure 5a shows the relationship between the axial force of the modified sample and the concentration of the cementation solution. It can be seen in Figure 5a that the compressive strength of the sample at a concentration of 1.25 mol/L was higher; it was twice that of the blank control group. Additionally, the received force was 2060 N, and the corresponding compressive strength reached 1.73 MPa. With the increase in concentration, the overall change trend of the compressive strength of the sample was to first increase and then decrease. Although the compressive strength value was larger at the concentration of 1.25 mol/L, when the concentration gradient was consistent, compared with the concentrations of 1.0 mol/L and 1.5 mol/L, the high-concentration cementing solution had a better improvement effect.

The samples were soaked and cured at a constant temperature of mixing; the samples were cured and mineralized in the cementation solution, and there were three samples in each group used to study the improvement effect of *Sporosarcina pasteurii* with different concentrations of cementation solution. As shown in Figure S1b, from left to right, the groups were the blank group and the 0.5 mol/L, 0.75 mol/L, 1.0 mol/L, 1.25 mol/L, and 1.5 mol/L groups, showing the differences in the cemented silt properties. It was observed that the samples with slightly lower cement concentrations were mainly damaged by local failure and transverse tensile failure. When the cement concentration reached 1.25 mol/L, although the samples were also damaged, they still maintained good integrity.

**Figure 6:**
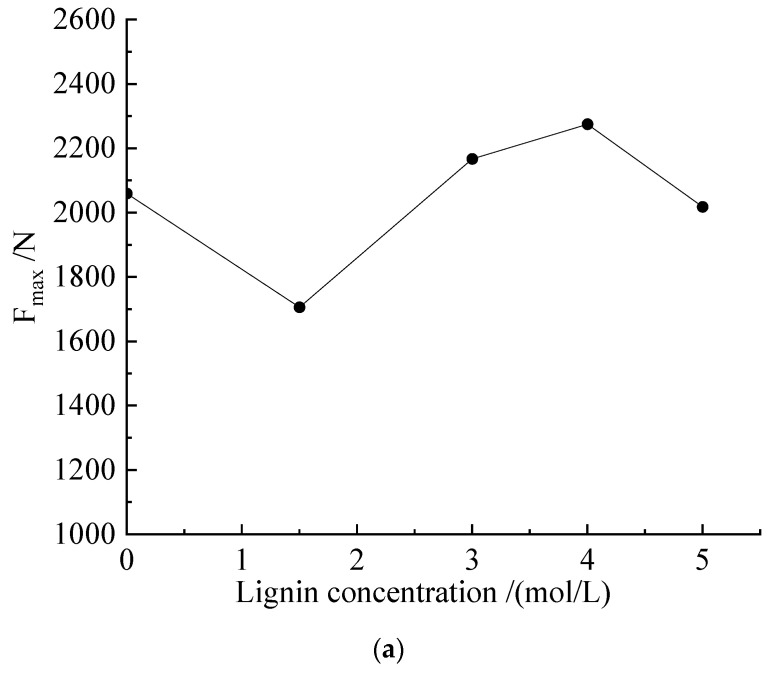

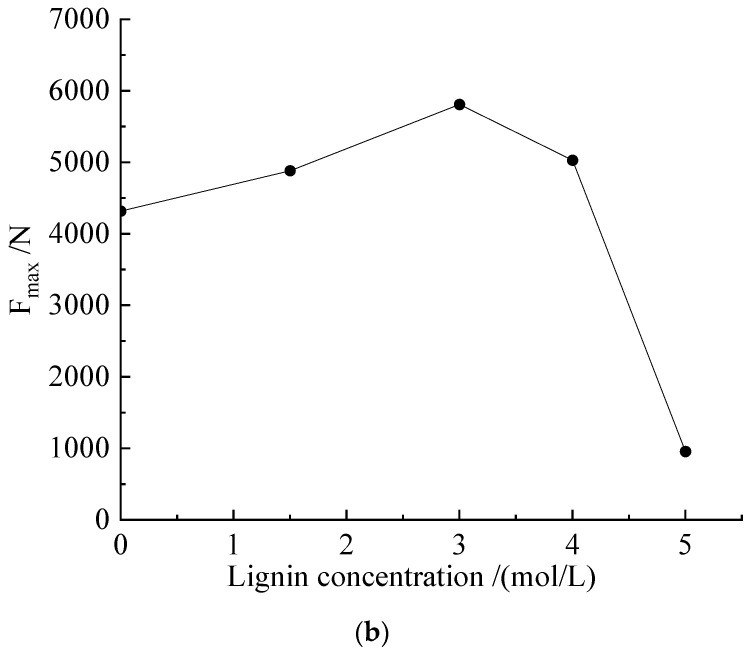
Lignin addition ratio and maximum axial force (F_max_) of M-L schemes of different samples. (**a**) Lignin addition ratio and maximum axial force (F_max_) of M-L scheme of constant-temperature sample. (**b**) Lignin addition ratio and maximum axial force (F_max_) of M-L scheme of soaked sample.

Compared with the samples obtained by the constant temperature and humidity curing, for the samples obtained by the constant-temperature immersion method, under the action of unconfined axial force, the failure mode was the same as before. The development trend was the same, and a better cementation effect was obtained when the cementation solution was 1.25 mol/L. Figure 6b shows the relationship between the axial force of the improved sample and the concentration of the cementation solution. The maximum axial force that the sample could withstand reached 4315 N, and the compressive strength was 3.62 MPa. However, except for the two concentrations of 1.25 mol/L and 1.5 mol/L, the compressive strength of the other concentrations decreased. It is speculated that the direct curing of the samples obtained by the punching method also had a certain strength and compaction. When the sample was immersed in the cementation solution, a small number of soil particles diffused into the cementation solution, and the internal structure of the sample was affected, which eventually led to a decrease in the compressive strength of the sample. When the concentration of the cementation solution reaches a certain value, the cementation effect is greater than the diffusion effect of soil particles, and the number of calcium ions involved in the reaction is much greater than in constant temperature and humidity maintenance, so it has a greater cementing effect.

Comparing the unconfined compressive strengths obtained using the two curing methods, the immersion method was found to have a stronger advantage. At the cementation concentration of 1.25 mol/L, the compressive strength of the samples obtained using the soaking method was much greater than that of the samples obtained using constant temperature and humidity curing.

#### 4.1.2. Analysis of Improvement Effect of MICP-Lignin (M-L) Scheme

After the 1.25 mol/L cementation solution interacted with *Sporosarcina pasteurii* and lignin, the failure shape of the specimen obtained using the constant temperature and humidity curing method under unconfined compression had a typical Z-shaped crack, as shown in Figure S2a. From left to right (a–d), the proportions of added lignin were 1.5%, 3%, 4%, and 5%. During the compression process, local failures and transverse cracks near the ends appeared in the group with a 5% lignin addition ratio; it can be seen that 5% lignin was not conducive to bonding between silt particles in this test. As shown in Figure 6a (the blank group is *Sporosarcina pasteurii* with a 1.25 mol/L cementation solution), for the samples obtained using the constant temperature and humidity curing method, when the lignin addition ratio was 4%, it had a good improvement effect, and the samples were able to resist a maximum axial force of 2275.5 N; the compressive strength was 1.9 MPa. According to the comprehensive analysis of the failure shape of the samples and test data, the addition of lignin was found to be beneficial for the improvement of the compressive strength of the samples, but it was not conducive to the improvement of the tensile properties of the samples.

The samples obtained using the constant-temperature immersion method are shown in Figure S2b. Under compression, the 1.5% lignin group and the 5% lignin group mainly showed tensile failure. Although the samples were damaged by compression, they maintained good integrity; this shows that the samples had a good cementation effect during the curing process. This was especially evident in two different post-curing test failure plots for the 5% lignin group. As shown in Figure 6b, via the mixed improvement of *Sporosarcina pasteurii*–cementum–lignin, for the sample cured using constant-temperature immersion with a 1.25 mol/L cementation solution, the addition of lignin at 1.5%, 3%, and 4% showed a good improvement effect, especially at 3% lignin. The axial force that the sample could withstand reached 5808.9 N, and the corresponding compressive strength was 4.84 MPa. When the ratio of added lignin reached 5%, the same trend appeared when lignin was improved alone (the same constant-temperature-soaking curing method). There was a significant drop in axial pressure.

### 4.2. Analysis of Unconfined Hydrostatic Strength Test Results

#### 4.2.1. Analysis of the Improvement Effect of the M Scheme

As shown in Figure S3, the sample cementation solution concentrations, from left to right, were 0.5 mol/L, 0.75 mol/L, 1.0 mol/L, 1.25 mol/L, and 1.5 mol/L. When the samples in this group were subjected to multi-level dynamic loads, they all showed drum-shaped deformation to different degrees; among these, oblique cracks appeared in the cementation solution concentration groups of 0.5 mol/L, 0.75 mol/L, 1.0 mol/L, and 1.5 mol/L; the 1.25 mol/L group had a more obvious X-shaped crack.

**Figure 7:**
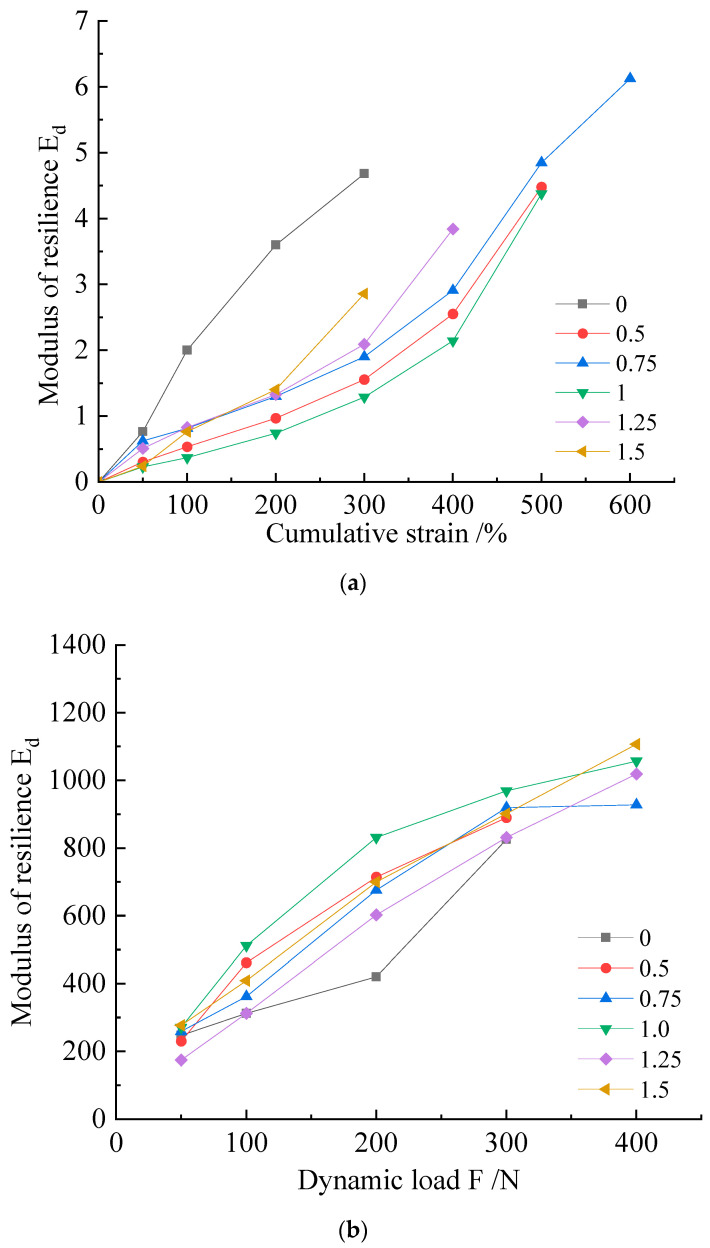
(**a**) Cumulative strain–resilience modulus (Ed) curves and (**b**) dynamic load–resilience modulus curves of M scheme samples.

The strain accumulation curve of the specimen under the action of a multi-level dynamic load is shown in Figure 7. The curves of the modified samples were all smaller than those of the blank control. It can be seen that the modified samples had better stress–strain relationships. Among these, the best improvement effect was found in the 1.0 mol/L cementation solution group. The curve obtained by the test generally had two stages: the linear stage and the plastic stage. When the loaded axial pressure was small, the sample was in a linear change stage, and the load and strain had a good corresponding relationship, close to the simple linear correspondence of F = k × x (F is stress, x is strain, and k is the coefficient); most of these critical values were 200 N. When the force on the sample reached a certain value, the slope of the curve changed rapidly, and these inflection points mostly occurred at 300 N. When the axial compression reached 400 N, the deformation of the specimen was more rapid.

Each level of dynamic load applied to the specimen corresponded to a different resilience modulus (Ed). Judging from the concentration of *Sporosarcina pasteurii* combined with different cementation solutions, the resilience modulus increased with the increase in dynamic load; the group with a 1.0 mol/L cementation solution concentration showed the best overall performance.

**Figure 8:**
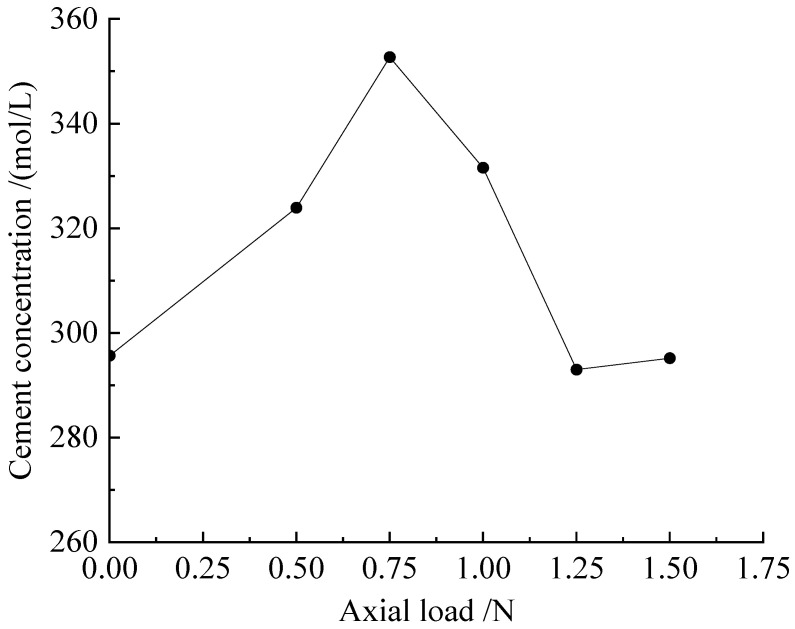
Samples of scheme M: F-plot of cementation solution concentration and axial load.

Figure 8 shows an analysis of the maximum axial load that each group of dynamic triaxial experiments could bear. When the concentration was 0.75 mol/L, the axial load of the sample was the largest, which was 352.74 N. Compared with the unconfined compressive strength of the corresponding dried samples, the measured values were 1245.8 N and 1140.7 N, which were much higher than the maximum axial pressure that the movable triaxial could withstand. This shows that the moisture content of the sample had a great influence on the mechanical properties of the sample. Although the trend of the curves measured by the two is the same, the difference with the unconfined curve is that the optimal cement concentration of the shuffle measured by the dynamic triaxial was smaller than the concentration measured by the unconfined test. According to the cumulative strain (ε), the resilience modulus (Ed), and the maximum axial pressure (Fmax) that the sample could withstand, the optimal cement concentration was found to be 1.0 mol/L or 0.75 mol.

#### 4.2.2. Analysis of Improvement Effect of MICP-Lignin Scheme

As shown in Figure S4, from left to right (a–b), the lignin addition ratios were 1.5%, 3%, 4%, and 5%. The samples with the combined action of *Sporosarcina pasteurii* and lignin did not have any obvious cracks when they reached the deformation failure standard, and the main deformation was drum-shaped.

**Figure 9:**
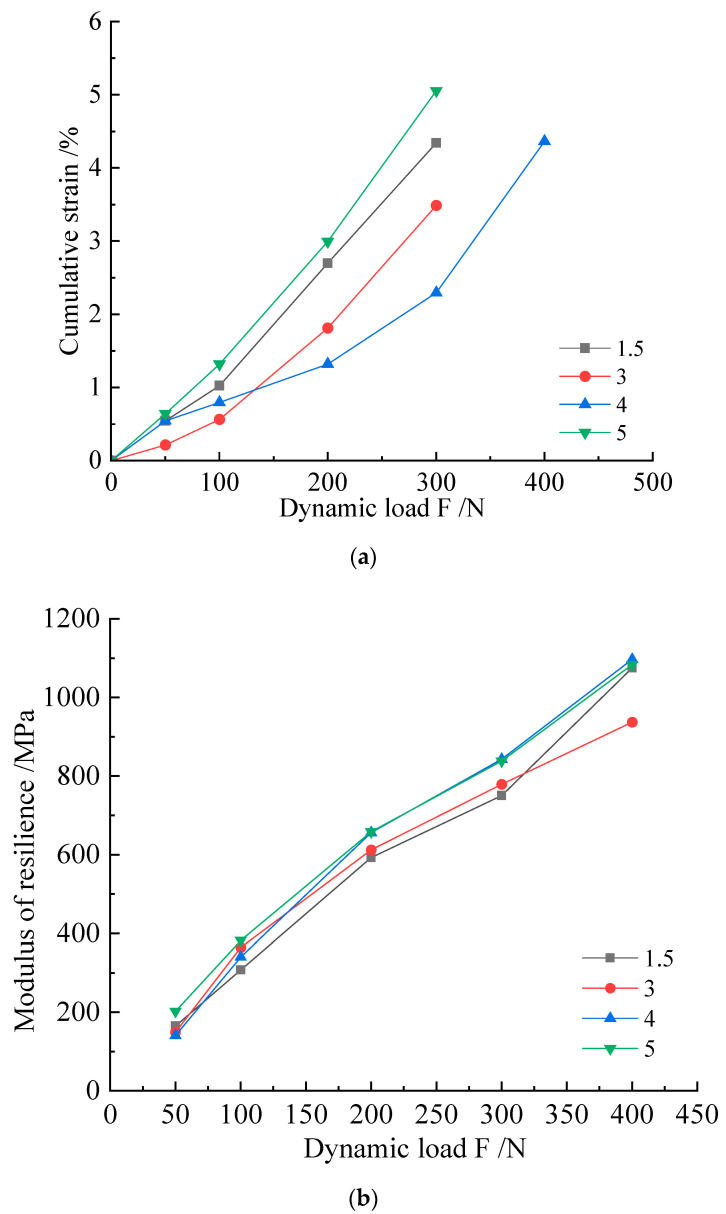
(**a**) Cumulative strain curves of M-L scheme specimens. (**b**) Resilience modulus (Ed) versus dynamic load curve.

As shown by the cumulative strain–dynamic load curve of this group, depicted in Figure 9, the best improvement was found in the 4% group. When the axial dynamic load reached 300 N, the cumulative strain of the 4% group was 2.3 mm, which was 3.5 mm and above for the other groups. From the perspective of the increase in or slope of the dependent variable at each stage, the order, from large to small, was 5%, 1.5%, 3%, and 4%. From the statistical resilience modulus, the samples exhibiting a high lignin content had a higher resilience modulus. Combined with the group using lignin to improve silt alone, the combination of MICP and lignin caused the optimal addition ratio of the sample to be increased. This phenomenon was mainly reflected in that the most common addition ratio of 3% was changed to 4%, and a resilience modulus of 4% or 5% was better. The specimens exhibiting this phenomenon did not show any relatively obvious fracture surfaces when they reached the specimen failure standard.

**Figure 10:**
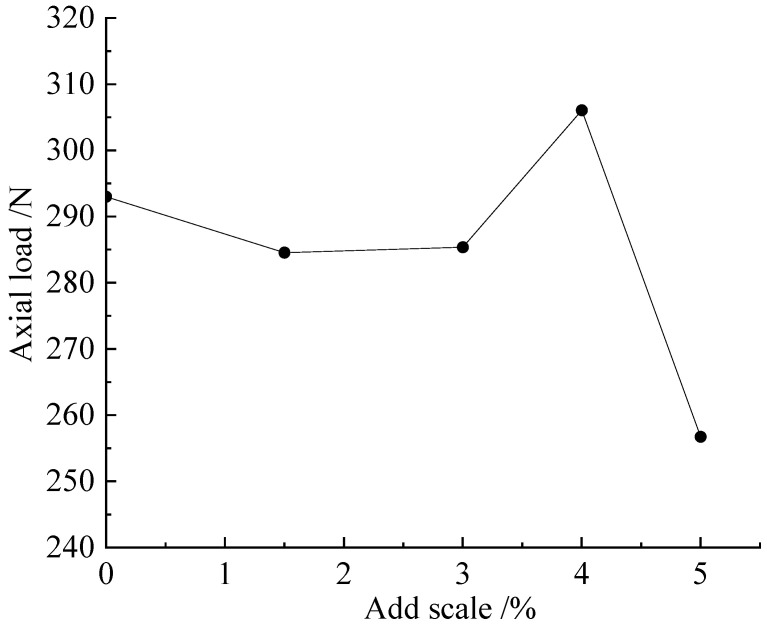
M-L scheme sample: ratio of lignin addition and axial load.

As shown in Figure 10, when the addition ratio reached 4%, the maximum axial load that the specimen could bear was 306.08 N. The addition of too much lignin led to a rapid decline in the sample’s performance (Figure 10). This phenomenon was consistent with the curve obtained by the constant-temperature immersion method in the unconfined compressive strength test. According to the comprehensive analysis of the cumulative strain curve, the resilience modulus, and the maximum axial load curve that the specimen could bear, in the improved method using MICP-n, lignin at 4% was found to be the optimal addition ratio.

## 5. Conclusions

This study assessed silt soil in the Beijing area. Various schemes, such as the use of MICP technology and lignin modification (alone or with mixed modifications of silt), were set up, and the improved performance was evaluated using unconfined compressive strength and dynamic triaxial tests.

- After the culture time of *Sporosarcina pasteurii* reached 24 h, the OD600 value was 1.7–2.0, and the activity value (U) was 930–1000 mM ms/min.
- The optimal concentration of the cementation solution for *Sporosarcina pasteurii* samples with constant-temperature immersion and constant temperature and humidity curing was 1.25 mol/L; among these, the compressive strength of the constant-temperature immersion sample was 3.62 Mpa, which was more than twice that of the constant temperature and humidity sample.
- For the samples of silt modified using MICP technology combined with lignin at a constant temperature and humidity, with the cement concentration of 1.25 mol/L, the optimal addition ratio of lignin was found to be 4%, and the compressive strength was 1.9 Mpa. The optimal addition ratio for the constant-temperature immersion sample was 3%, and the compressive strength was 4.84 MPa.
- Under the action of a confining pressure of 50 kPa and multi-level cyclic loads, in the scheme of improving silt using *Sporosarcina pasteurii* and different concentrations of cementation solution, 1.0 mol/L was found to be the optimal concentration of cementation solution. In the experimental scheme of a 1.25 mol/L cementation solution plus *Sporosarcina pasteurii* combined with lignin, 4% was the optimal ratio of lignin addition.

## Figures and Tables

**Table 1:** Basic physical parameters of silt.

Soil Sample Name	Air-Dried Moisture Content W/%	Liquid Limit WL/%	Plastic Limit WP/%	Plasticity Index IP	Grain-Specific Gravity GS	Dry Density ρ_d_/g·cm^−3^	Density ρ/g·cm^−3^
Silt	0.682%	25.30	16.71	8.98	2.63	1.577	1.921

**Table 2:** Statistics of the number of samples made.

Concentration (mol/L)	Bacterium + n Cementation Liquid	Add Scale/%	Bacteria + n Lignin (1.25 mol/L Cementing Solution)
0.5	5	1.5	5
0.75	5	3	5
1	5	4	5
1.25	5	5	5
1.5	5	6	0

**Table 3:** Compositions of cementation solutions with different concentrations.

Concentration/mol·L^−1^	0	0.5	0.75	1.0	1.25	1.5
Anhydrous calcium chloride/g	0	55.49	83.24	110.98	138.73	166.47
Urea/g	0	30.03	45.05	60.06	75.08	90.09

## Data Availability

All data that support the findings of this study are available from the corresponding author upon reasonable request.

## Supplementary Materials

The following supporting information can be downloaded at https://www.mdpi.com/article/10.3390/microorganisms11020281/s1, Figure S1: The pressure failure diagrams of different samples for the M scheme. (A) The pressure failure diagram of the constant-temperature sample for the M scheme. (B) The pressure failure diagram of the soaked sample for the M scheme; Figure S2: Compression failure diagrams of different samples in the M-L scheme. (A) The pressure failure diagram of the constant-temperature sample in the M-L scheme. (B) The compression failure diagram of the soaked sample in the M-L scheme; Figure S3: Failure diagram of M scheme specimen under dynamic load; Figure S4: Failure diagram of M-L scheme specimen under dynamic load. Table S1: Particle size ratio and coefficient. Table S2: The amount of each substance consumed in preparing a single sample. Table S3: Relationship between lignin addition ratio and Fmax.
