# Supplementary material for: Experimental Study on Silt Soil Improved by Microbial Solidification with the Use of Lignin
Source: Microorganisms. 2023 Jan 20;11(2):281. doi: 10.3390/microorganisms11020281 (PMC9965713; doi:10.3390/microorganisms11020281)
Supplement: Supplementary file 1 [file microorganisms-11-00281-s001.zip › microorganisms-2082677-supplementary.pdf]

Supplementary materials

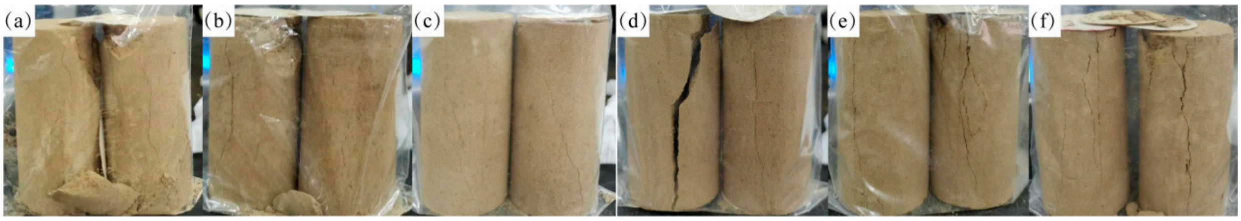

(A) The pressure failure diagram of the constant temperature sample for the M scheme.

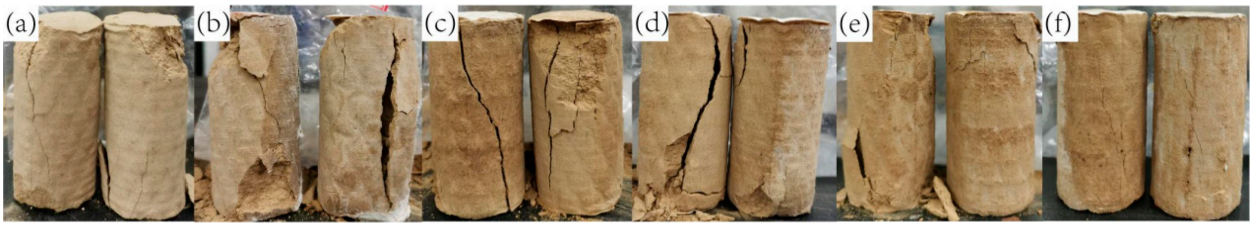

(B) The pressure failure diagram of the soaked sample for the M scheme.

Figure S1. The pressure failure diagram of different samples for the M scheme; (A) The pressure failure diagram of the constant temperature sample for the M scheme;(B) The pressure failure diagram of the soaked sample for the M scheme. The cement concentration of the sample from left to right is: (a)0.5mol/L, (b)0.75mol/L, (c)1.0mol/L, (d)1.25mol/L, (e)1.5mol/L.

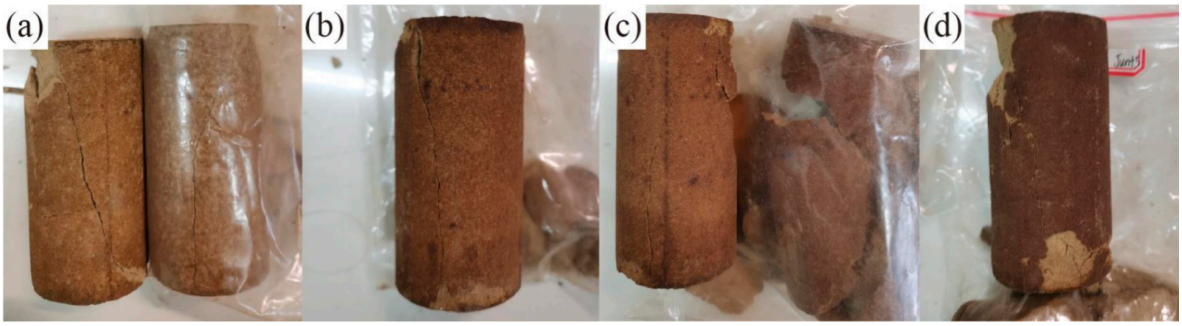

(A)The pressure failure diagram of the constant temperature sample in the M-L scheme.

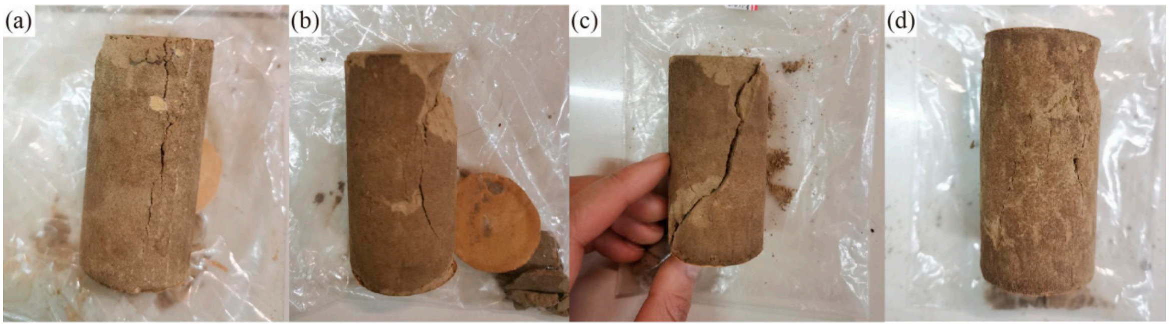

(B) Compression failure diagram of soaked sample in M-L scheme.

Figure S2. Compression failure diagram of different samples in M-L scheme; **(A)**The pressure failure diagram of the constant temperature sample in the M-L scheme**;** **(B)** Compression failure diagram of soaked sample in M-L scheme. From left to right, the lignin addition ratio of the sample is: (a)1.5%, (b)3%, (c)4%, (d)5%.

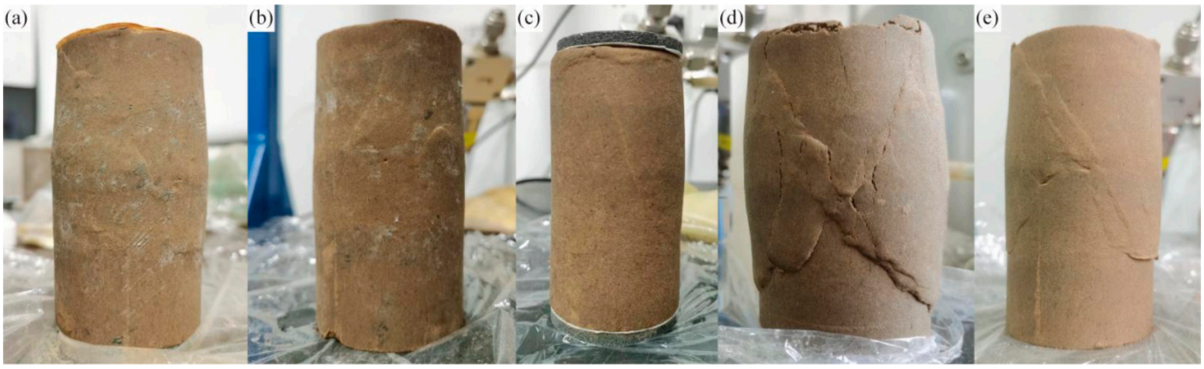

Figure S3. Failure diagram of M scheme specimen under dynamic load. The cement concentration of the sample from left to right is: (a)0.5mol/L, (b)0.75mol/L, (c)1.0mol/L, (d)1.25mol/L, (e)1.5mol/L.

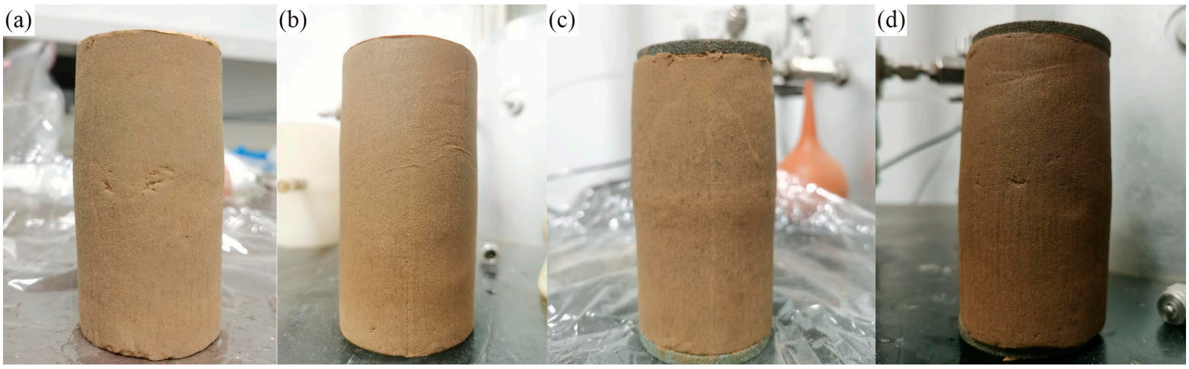

Figure S4. Failure diagram of M-L scheme specimen under dynamic load. From left to right, the lignin addition ratio of the sample is: (a)1.5%, (b)3%, (c)4%, (d)5%.

Table S1. Particle size ratio and coefficient.

d60	d50	d30	d10	Cu	Cc
0.033	0.027	0.014	0.004	8.25	1.485

Table S2. The amount of each substance consumed in preparing a single sample.

	Bacterial liquid	Soil	cementing solution
Unlimited	48mL	160g	48mL
three-axis	96mL	320g	48mL

Table S3. Relationship between lignin addition ratio and Fmax.

The proportion of lignin added/%	0	3	6	9	12	15
F_{max}/N	1017	2489.9	1401.65	1173.28	925.9	829.8
